# Homicide—Suspected Simulation of Insanity

**Published:** 1874-12

**Authors:** I. Ray


### Selections.

*Homicide—Suspected Simulation of Insanity.* By I. RAY, M.D.

No matter connected with insanity makes a larger draft on the resources of the expert, than the task of deciding correctly in some cases of suspected simulation. This might naturally be expected where a sane person is performing his part with all the ingenuity and cunning which a life of criminal habits is apt to develop ; but the difficulty is often none the less where the manifestations are very demonstrative and genuine ; and the reason is, that however these may conflict with the results of one's own observations, no one profoundly impressed with a sense of the infinite diversity of nature, even in her wanderings, will be in haste to conclude that they are, on that account, simulated. And the chances of reaching a correct conclusion are not increased by the disposition of the expert to forget that, in the very act of guarding against the deceptions of the patient, he is very apt to deceive himself. To add to his embarrassments, the opportunities for testing the mental condition may be very limited and quite unsuitable. A few interviews with the party in his cell, with such information as attendants may give, furnish, perhaps, the only materials with which he must construct his final opinion. That they are often insufficient to warrant any stronger conclusion than a guess, is precisely what might be expected, and the fact does no discredit to the sagacity of the expert. And yet this is not incompatible with the other fact, that under the surveillance of a Hospital for the Insane, the true character of a suspicious case cannot fail, at last, of being correctly understood by any one of considerable clinical experience and tolerable sagacity.

I have thought the following case worth relating, because it presents a rather unusual combination of traits, and, for that reason, may convey some useful hints to future observers.

In March, 1871, Michel Trimbur, with two or three other young men, was convicted, in one of the courts of Philadelphia, of a heinous outrage upon a young woman, and sentenced to fifteen years imprisonment in the Eastern penitentiary. He was of Irish parentage, about twenty-two years old, of rather small stature, and with a countenance indicative neither of stupidity nor ferocity. The crowded condition of the prison rendering it necessary, to some extent, to put two in a room, T. and one of his associates in crime were assigned to the same room, with their mutual consent and satisfaction. And up to the last, they seemed to be on the best of terms with each other. On the morning of the 7th of May, 1872, when tne keeper went to their cell with their breakfast, Michel said very quietly that Webb would not need any. On inquiry, Webb was found on the bed quite dead, with his head badly bruised. Michel immediately confessed that he killed him while lying in bed, with the board used for closing the ventilator. When asked how it happened, he said he rose early, and struck Webb on the head, several times, he making no resistance. When asked for his reasons, he admitted that they had had no quarrel, but said that Webb had frequently abused his (T.'s) mother, and he would not allow anybody to do that. Neither then nor subsequently did he express any sorrow for what he had done.

Previous to his trial for this second crime, his counsel requested me to ascertain his mental condition, as there was some reason for suspecting that T. was crazy. The officers of the prison where I went for this purpose, informed me that no indications of insanity, before the murder, had been noticed by any one, and that they first heard of it some time afterwards. On being asked what they had heard, I was told, that while passing from one part of the prison to another, he looked upwards and said to the keeper, "O, what bright thing is that up there?" meaning the sun; and also, that having made considerable noise during the night, he was threatened with some privation if he persisted in it, and accordingly he became quiet from that night forth. The officers regarded these two manifestations as mere make-believe, and had no faith in his pretended insanity. During my first visit, which was over one hour long, we conversed about the circumstances of his two crimes, about his family, his education and employment, and about ordinary affairs and topics in which he might be supposed to be interested, and while he talked freely, without the least reluctance, not a word fell from his lips that could excite a suspicion of insanity. Though not particularly deficient in understanding, he had obviously received but little education, had no regular employment, and his social surroundings had been of a pretty low order. He gave the same account of the murder which he did at first, and without any sign of regret. Of the first offense, he then and always subsequently declared his innocence, and there was some reason to think, that in this he spoke the truth, his only offense being that of keeping bad company.

A few days afterwards, I made him another visit. For some fifteen minutes the conversation was very much like that of the previous visit. Then he rather suddenly went to the farther side of his cell, and took from a shelf a cheap card photograph of a child, or young girl, which he brought to me, his whole manner exhibiting a sort of earnestness which I had not observed before. This, he said, was his mother, the Virgin Mary, and forthwith he launched out into a stream of talk, consisting of wild, strange, incongruous notions, connected by no obvious bond of association. In this jumble of matter, in uttering which he needed no prompting, there was no repetition of words or phrases, and it was curious to see how a person who could not be supposed to have gained much command of language, could vary his modes of expressing such barren nonsense. The following specimen of his talk will give a better idea of it than any description possibly can:

"I give all of it to my mother. Neither is jealous, and she shall be my wife. I am going to be most a spirit. She needn't die for me and live as long as I live. She shall have as much to do with everything as I do. She is Mary the mother. I am her third husband—will give her my writings. Jesus Christ was two or three hundred when he died. He laid right down and died. Nothing comes into this world with stronger eyes than I have. I have got to die for that person—any strong person, Lazarus. I am Lazarus. I am to have the strongest eyes in the world, the best hair, the best of everything. Prettiest man that ever was born—made of the best stuff. Miss Wills was Moses' second wife. Mary, the mother of Jesus, is to be my wife."

In the course of the next two months I visited him twice, and obtained only a repetition of the same sort of discourse and behavior; and these were the materials, in connection with the facts already mentioned, derived from the officers of the prison, out of which I was to form my opinion respecting the prisoner's mental condition; and the task was not an easy one.

Unquestionably, his exclamation about the sun was simulation. In no form of mental disease with which he could possibly have been affected, can we suppose such a lesion of the mental faculties as that would imply. He was not demented; he was not raving; he had no hallucinations; his perceptive powers were neither weakened nor perverted; and it was the only instance of the kind. This fact raised the suspicion of simulation in regard to all his crazy manifestations, some of which, certainly, were not calculated to remove that suspicion, though none were obviously incompatible with insanity. The entire correctness of his discourse in the first interview, the abruptness of the transition into a stream of wild and whirling words, and the peculiar character of the thoughts they conveyed, produced a first impression not very favorable to a conviction of his honesty. The last mentioned trait seemed indicative of a low grade of dementia more than any other form of insanity, and this seemed to be inconsistent with the clearness and vigor of the rest of his discourse. During my first visit, he was not aware of my function or purpose, but at my subsequent interviews, he knew, at least, that I was a physician. This fact, too, was a ground of suspicion, stronger to others, probably, than to myself. So far, then, the tendency of his manifestations favored the theory of simulation.

On the other hand, a mature consideration of all the circumstances of the case convinced me that they presented nothing incompatible, necessarily, with the existence of real insanity. Men who have been much conversant with the insane in hospitals—not meaning those whose knowledge consists in having *seen* many thousand patients—need not be told that sometimes, for one purpose or another, they make a show of being more crazy than they really are. They see what is going on around them, and, if not too much occupied with their own condition, they need only a little power of mimicry and a sufficient inducement, to imitate it. In one of our regular hospitals—many years ago, thank God—it was one of the customary performances for the entertainment of visitors, to show off a big, double-fisted, unkempt, unshorn patient, acting the role of the furious madman. And he did it, if not very correctly, yet well enough to excite the applause of the lookers-on.

Many insane do certain things as well as they ever did; they plan, contrive, anticipate, in furtherance of a special purpose. There is nothing strange or anomalous, therefore, in the fact of their endeavoring to act the madman; of course, in a phase of the disease different from that which they really exhibit. The criminal classes, to which most of these simulators belong, know, as well as everybody else, that the plea of insanity is one of the dodges whereby people now seek to escape the punishment of their crimes, and they may not forget it, nor neglect to act accordingly, when they become insane themselves. It may seem, at first glance, a work of supererogation for a man obviously insane to endeavor to impress others with the belief that he is the subject of another and even a very different kind of insanity. The mystery vanishes when we consider that usually no insane person thinks himself to be insane, believing, as they do, with extraordinary tenacity, that the delusions they entertain are gospel truths; while the folly of their actions is strictly in accordance with the requirements of the occasion, and both the delusions and the folly indicative, as they think, of the soundest reason. For the purpose in view, therefore, the role of the simulator is as necessary to them as to others. Thus, Trimbur, being unconscious of his own real insanity, but with mind enough to understand his situation, and to remember what he had heard about insanity in connection with crime, concluded to make a show of being crazy.

I have said that the style of his discourse resembled that of dementia—a form of disease not to have been expected under the circumstances—and therefore suspicious. Unusual, such a phenomenon certainly is, but I am not prepared to say that it is conclusive proof of simulation. We must be cautious how we consider the experience of any individual in so vast a field as the aberrations of the human mind, as having comprised every form and shape which they can possibly take. The larger our experience, the more are we impressed with the infinite diversity of nature, even in the domain of mental pathology, and all, too, under the control of inflexible law. A closer observation of the prisoner might have revealed some connecting links between the sound and unsound trains of thought, which would have rendered the contrast less extraordinary.

Again, it was a very significant fact, in regard to his mental movements, that the perceptive faculties exhibited no aberration whatever, except the single instance first mentioned. Questions involving relations of time, space, distance, etc., were always correctly answered, and, indeed, in regard to all ordinary matters, clearly beyond the sphere of his wild notions, there was not a sign of derangement, real or pretended. Thus he abstained altogether from the dodge—if I may use the term in lieu of a better—which naturally presents itself to the simulator. His object is to make an impression, and he chooses, therefore, the strongest and least mistakable manifestations of disease—such as will be known at once to all men. He says, perhaps, that he is a hundred years old; that a place near by is a dozen miles off; that he knows not at all, persons with whom he has been intimate all his life; and, in short, he misrepresents the simplest matters of fact that happened under his observation. He may not show this trait in every particular, because he may be afraid of over-acting his part, and think his purpose better answered by holding up occasionally, but he will not entirely abstain from using the opportunity thus afforded for making an impression.

It must be borne in mind, that it is the simulator's purpose—the part he has undertaken to perform—to appear insane, and consequently he is careful not to appear sane, especially on occasions when his ends would be furthered by a successful imitation. He even lays plans for obtruding his mental disorder on the notice of the observer, though exercising all his ingenuity, perhaps, to conceal his design. It is not very likely, therefore, that Trimbur, if he had shrewdness enough to undertake the task of convincing others that he was actually insane, would have allowed our first interview to pass without the slightest attempt in that direction. He might not have suspected my purpose precisely, but he could scarcely have helped suspecting that I had some purpose. Accompanied, as I was, by the physician of the prison, by his keeper, and part of the time by one of the officers, it was an opportunity he would have been only too glad to use. Indeed, with the exceptions above mentioned, it did not appear that he exhibited any sign of insanity, simulated or otherwise, to any officer of the prison.

It is a circumstance also worth considering, that in his statements respecting the murder, when first discovered, and before he was likely to have formed any plan of simulation, he made no attempt to palliate the deed, as a sane man probably would. He never pretended that there was any sudden quarrel between him and his companion, that the latter provoked and assaulted him, and that he acted in self-defense. Calmly and quietly he declared that he killed him while lying in bed asleep, because he had abused his mother.

We are to bear in mind that, on any question of simulation, we are to take into the account the moral, intellectual and social character of the party. A skillful attempt implies some knowledge of men and things, some notions, however crude, about insanity, some power of mimicry, and great tenacity of purpose. Most simulators have picked up from books or personal observation, some ideas of how insane people act and talk; and sometimes a knowledge of their antecedents will show the particular model on which their imitation is founded. Now, no mortal could have been less qualified for acting the simulator of insanity than Trimbur. He was young, not very bright, with little education, his knowledge of the world was limited to a particular locality, abounding in the rough and rowdy element, and he probably had never seen an insane person in all his life. I found it difficult to believe that a person with such a record, could, by pure force of invention, achieve a jumble of thoughts so nearly resembling the utterances of the insane. And in recording them, as in the specimen above given, I found it impossible to follow him so rapidly as to get the vague, broken, indistinct, half-uttered expressions marking the transitions from one definite thought to another, and of which only those familiar with the insane can have any adequate conception.

Here, then, were the reasons for and against the theory of deception, and had no attempt to deceive been made, as I was obliged to think there had, I should not have hesitated to believe him truly insane. As it was, they greatly preponderated, but so long as there was wanting that test furnished by a more close and continuous observation of discourse, deportment, manners, freaks and fancies, than is implied in an occasional, formal interview, when the person is controlled and restrained by the presence of a stranger, and if actually attempting to deceive, summons all his resources for the occasion—such an observation as he would undergo in the wards of a Hospital for the Insane—I was not ready to relinquish every doubt. However strong the presumption in favor of the prisoner, complete confidence was unwarranted, under the circumstances. And so on the trial, in September, 1872, I testified, that while the prisoner had every appearance of being insane, he might possibly be simulating, and that I had not had such opportunities for testing his mental condition as were needed for forming a certain conclusion. He was convicted of murder in the second degree, but his counsel applied for a new trial, which, after considerable delay, was granted. On the 14th of November, 1873, he was tried again, and acquitted, on the ground of insanity. The medical officers and others connected with the prison had become so strongly convinced of his insanity, that their opinion was readily accepted by the jury.

In May last, I visited Trimbur in the jail. He had grown more stout in body, and more stolid in look. The keepers told me he had been uniformly quiet and well behaved, and neither exalted nor depressed. He was as ready to talk as ever, and his talk was the same sort of jargon that I heard before. He spoke occasionally of the Virgin Mary, though less of her being his wife or mother, but his favorite topic was the excursions he frequently made into various parts of the city. Among those who came to his cell, at one time or another, and took him out, he mentioned General Washington, General Grant, Adam Sharp, Mr. Down, "or some one just as they are sent from Heaven or court. The Virgin Mary sometimes, because she is my aunt." When asked what he did when out, he replied that he kept a shop, and sold things, and again to the same question, he replied, "you must do something to help Philadelphia." Running on in his way, he said, "Those people are all mixed up, all help get the world in order. When they get arrested, I make it very easy for them, I would like to get out of prison. Most all of them is in use—the best of them. Most in prison don't get out." The indistinct utterances, the rambling undertones interspersed among the more definite expressions, so characteristic of insanity, it is beyond my power to give. Again, about the middle of August, I visited T., and found no change in his manifestations. If stopped in the midst of his jargon, and asked how old he was, how many brothers and sisters he had, his mother's maiden name, how far it was from a certain locality to a certain other, the names of some old associates, the answers were correctly given.

About this time I first heard of an incident, which, had I known it in the beginning, might have removed my doubts respecting the true character of the case. It seems that a week or two before the murder, T. was visited by his mother, who came away greatly distressed, saying to one of the keepers, that her son talked very strangely, as he had never talked before, and she was afraid he was getting crazy.

The history of the case here related, justifies me, I think, in drawing the following conclusions, viz: first, that Trimbur is now, and was at the time of the homicide, really insane; secondly, that apprehending the consequences of the act, he concluded to simulate the disease, of which he was already the unconscious subject; and thirdly, that finding it produced no impression, or that his powers of deception were unequal to the task, he abandoned the attempt after one or two trials.

The above narrative furnishes a strong illustration of the difficulty of dealing with such cases in penitentiaries and jails. No one practically acquainted with the ways of the insane, need be told that such institutions furnish very inadequate opportunities for ascertaining the real mental condition of a prisoner suspected of feigning insanity. So far as it is confined to the manifestations during a special interview, he may escape detection, because he is fairly pitted against the expert, and the trial of his powers is very brief. In a hospital, on the contrary, he is observed much of the time, in every variety of circumstances, and liable at any time to be watched when he least suspects it. His manners, gestures, looks, habits, temper, conversation, may yield revelations more satisfactory than the wildest utterances; and the show of disease so well maintained by a careful and special effort, is constantly liable, under the influence of familiar circumstances, to slide into a natural deportment. For lack of any opportunity of this kind, I was obliged to withhold a positive opinion, notwithstanding my strong impression that I was dealing with a case of genuine insanity. As it happened, no harm was done, for the court, understanding the situation, purposely forbore to press the hearing for a new trial, the prisoner in the meantime becoming so obviously insane, that his acquittal was not resisted by the District Attorney. Every court, however, is not so considerate, and we can easily believe that in some parts of our country, Trimbur would have been summarily hanged.

It is highly desirable that the opportunity should always be furnished somehow, for subjecting such cases to a kind of examination long enough and close enough to remove every doubt. Some of them, surely, might be satisfactorily investigated, even in the prisons, if the physicians attached to those institutions had some practical knowledge of the disease, and it were made their duty, as it never is now, to examine into their mental condition, for the purpose of giving an opinion on the trial. Why should it not be an indispensable qualification for the office, that they should be familiar enough with mental affections to entitle their opinions to some degree of respect?—*Am. Jour. of Insanity, Oct.* 1874.

